# *Asparagus officinalis* combined with paclitaxel exhibited synergistic anti-tumor activity in paclitaxel-sensitive and -resistant ovarian cancer cells

**DOI:** 10.1007/s00432-022-04276-8

**Published:** 2022-08-25

**Authors:** Xin Zhang, Jiandong Wang, Yali Fan, Ziyi Zhao, Sarah E. Paraghamian, Gabrielle M. Hawkins, Lindsey Buckingham, Jillian O’Donnell, Tianran Hao, Hongyan Suo, Yajie Yin, Wenchuan Sun, Weimin Kong, Delin Sun, Luyu Zhao, Chunxiao Zhou, Victoria L. Bae-Jump

**Affiliations:** 1 Department of Gynecologic Oncology, Beijing Obstetrics and Gynecology Hospital, Beijing Maternal and Child Health Care Hospital, Capital Medical University, Beijing, 100026 People’s Republic of China; 2 Division of Gynecologic Oncology, University of North Carolina at Chapel Hill, 170 Manning Dr, Chapel Hill, NC 27599 USA; 3 Division of Gynecologic Oncology, Lineberger Comprehensive Cancer Center, University of North Carolina at Chapel Hill, 450 West Dr, Chapel Hill, NC 27599 USA; 4 Shandong Juxinyuan Asparagus Industry Development Research Institute, HeZe, 274400 Shandong People’s Republic of China; 5 Shandong Juxinyuan Agricultural Technology Co. LTD, HeZe, 274400 Shandong People’s Republic of China

**Keywords:** *Asparagus officinalis*, Paclitaxel resistance, Synergy, Ovarian cancer, DNA damage, Cytotoxicity

## Abstract

**Purpose:**

Although paclitaxel is a promising first-line chemotherapeutic drug for ovarian cancer, acquired resistance to paclitaxel is one of the leading causes of treatment failure, limiting its clinical application. *Asparagus officinalis* has been shown to have anti-tumorigenic effects on cell growth, apoptosis, cellular stress and invasion of various types of cancer cells and has also been shown to synergize with paclitaxel to inhibit cell proliferation in ovarian cancer.

**Methods:**

Human ovarian cancer cell lines MES and its PTX-resistant counterpart MES-TP cell lines were used and were treated with *Asparagus officinalis* and paclitaxel alone as well as in combination. Cell proliferation, cellular stress, invasion and DMA damage were investigated and the synergistic effect of a combined therapy analyzed.

**Results:**

In this study, we found that *Asparagus officinalis* combined with low-dose paclitaxel synergistically inhibited cell proliferation, induced cellular stress and apoptosis and reduced cell invasion in paclitaxel-sensitive and -resistant ovarian cancer cell lines. The combined treatment effects were dependent on DNA damage pathways and suppressing microtubule dynamics, and the AKT/mTOR pathway and microtubule-associated proteins regulated the inhibitory effect through different mechanisms in paclitaxel-sensitive and -resistant cells.

**Conclusion:**

These findings suggest that the combination of *Asparagus officinalis* and paclitaxel have potential clinical implications for development as a novel ovarian cancer treatment strategy.

**Supplementary Information:**

The online version contains supplementary material available at 10.1007/s00432-022-04276-8.

## Introduction

Ovarian cancer (OC) is the most lethal gynecologic cancer in women and the fifth leading cause of cancer death among women the United States. It is projected that there will be approximately 19,880 new cases and 12,810 cancer-related deaths in the United States in 2022 (Siegel et al. 2022). Since most early-stage OCs lack disease-specific symptoms, approximately 75% of women are diagnosed with OC at advanced stages, presenting numerous treatment challenges due to metastasis, recurrence, and acquisition of progressive chemoresistance, leading to 5-year survival rates for OC patients with stage 3 and 4 disease of only 42% and 26%, respectively (Jayson et al. 2014; Tymon-Rosario et al. 2021). Thus, there is an urgent need to develop new therapeutic approaches for advanced and recurrent OC (Tymon-Rosario et al. 2021; Pereira et al. 2021).

Given that paclitaxel (PTX) as a single agent has shown favorable response rates in patients with persistent or recurrent disease, PTX has emerged as one of the most active agents in OC clinical trials (Mosca et al. 2021; Tendulkar and Dodamani 2021; Tropé et al. 1997). The combination of PTX with a platinum analogue results in a significant improvement in response and survival in patients with advanced/recurrent disease, and thus represents our first-line treatment regimen (Pokhriyal et al. 2019; Hoskins et al. 2010). However, OC cell acquired resistance to PTX is one of the leading causes of treatment failure and death in OC patients, ultimately limiting the overall benefits of this treatment (Tymon-Rosario et al. 2021). As PTX-resistant patients lack effective alternatives to PTX, identifying ways to increase PTX susceptibility and reduce PTX resistance would be an important advance in OC (Mosca et al. 2021; Das et al. 2021).

Natural products have been identified as sources of medicines for various human diseases. More than 3000 plants have been documented to be effective in the treatment of cancer, of which more than 600 natural compounds have potential anti-cancer effects in multiple preclinical models (Acquaviva et al. 2022; Muhammad et al. 2022). Bioactive compounds isolated from natural products with pharmacological properties may contain anti-tumor effects with little or no side effects (Subramaniam et al. 2019). Several FDA-approved natural compounds of plant origin such as colchicine, etoposide, and PTX have become clinically well-known antineoplastic drugs (Muhammad et al. 2022; Tan et al. 2021). *Asparagus officinalis* (ASP) is a type of liliaceous perennial vegetable crop that has been shown to possess numerous biological activities including anti-tumorigenic, anti-oxidant, anti-fungal, anti-inflammatory and immunomodulatory effects (Xu et al. 2021a; Zhang et al. 2018a; Wang and Ng 2001; Romani et al. 2021). The main bioactive constituents of ASP are a group of phytochemicals including flavonoids, steroidal saponins and polysaccharides. Phytochemicals extracted from ASP have been identified as having significant anti-tumorigenic potential in vitro and in vivo (Romani et al. 2021; Zhang et al. 2020, 2021a; Xiang et al. 2014; Wang et al. 2013). Importantly, ASP potentiated the anti-tumorigenic effects of mitomycin in hepatocellular carcinoma cells and a mouse xenograft model of hepatocellular carcinoma (Xiang et al. 2014). Our previous research found that ASP exhibited anti-proliferative and anti-metastatic effects in OC cells and a transgenic mouse model of OC, and that ASP combined with PTX had synergistic anti-proliferative activity in OC cells (Xu et al. 2021b). Thus, our current study aims to evaluate the possible anti-tumorigenic effects of the ASP extract in combination with PTX on cell proliferation, apoptosis, cellular stress and invasion in human PTX-resistant and -sensitive OC cell lines.

## Materials and methods

### Cell culture and reagents

The human OC cell lines MES and its PTX-resistant counterpart MES-TP were gifts from Dr Sikic (Stanford University School of Medicine). MES-TP cell line was developed paclitaxel combined with the P‐glycoprotein inhibitor PSC833. MES-TP cells were 4.6 times more resistant to PTX than MES cells (Moisan et al. 2014). Both cell lines were grown in McCoy’s 5A (Thermo Fisher Scientific, Waltham, MA) containing 10% fetal bovine serum, and penicillin (100 U/ml) and streptomycin (100 U/ml) in a constant temperature environment of 37 °C and 5% CO_2_. For the MES-TP cell line, 10 nM of PTX was added to the media. All antibodies used in this study were purchased from Cell Signaling Technology (Danvers, MA) and ABclonal (Woburn, MA). DMSO, PTX, MTT, crystal violet, DCFDH-A, JC-1 and Laminin were from Sigma (St. Louis, MO).

### Preparation of ASP extract

The ASP extract was supplied by the Shandong Shuoyi Biotechnology Co, LTD, P.R. China. The shoots of asparagus officinalis (ASP) were used to prepare the ASP extract. The procedure followed the protocol of our previous publication (Xu et al. 2021a). The ASP extracts were tested by the local government agency for 11 pesticide ingredients. No pesticide components were found in the extracts (Supplemental Table 1 and Table 2).

### Cell proliferation assay

The MES and MES-TP cells were cultured in 96-well plates with 4000 cells/well. After 24 h of incubation, the cells were treated with ASP, PTX or the combination for 72 h, and then 5 ul MTT (5 mg/ml) was added to each well for 1 h at 37 °C. The cells were then lysed with 100 ul DMSO/well, and the absorbance at 575 nm was measured using a microplate reader (Tecan, Durham, NC). The IC50 value for ASP and PTX was then calculated by the IC50 Calculator (AAT Bioquest, Sunnyvale, CA).

### Colony formation assay

The cells were plated at a density of 100/well in 6-well plates containing a standard culture media overnight, and then treated with 0.1 mg/ml ASP, 1 nM PTX or the combination for 24 h. The cells were fed every 3 days with fresh media for up to 12 days. The colonies were then fixed with 4% formaldehyde (Thermo Fisher Scientific) and stained with 0.1% crystal violet. Colonies containing 50 cells or more were counted under a Thermo Scientific Invitrogen EVOS microscope.

### Cleaved caspase 3, 8 and 9 assays

The MES and MES-TP cells were cultured at 2.5 × 10^5^ per well overnight, and then treated with 0.5 mg/ml ASP, 5 nM PTX and the combination for 14–16 h. The cells were washed with PBS twice, and 200 ul lysis buffer was then added into each well. The BCA assay (Thermo Fisher Scientific) was used to measure the concentration of lysis buffer. Reaction buffer, containing caspase 3 or caspase 8 or caspase 9 substrates (AAT Bioquest), was added to the lysis buffer in black 96-well plates at 37 °C for 20 min. The fluorescence intensity of cleaved caspase 3, caspase 8 and caspase 9 activity was detected by a Tecan microplate reader. These assays were repeated three times for consistency of results.

### Reactive oxygen species (ROS) assay

The cells were seeded at 8000 cells/well in 96-well black plates overnight, and then treated with 0.5 mg/ml ASP, 5 nM PTX, or the combination for 8 h to induce ROS. DCFDH-A (20 uM) was added to each well and incubated for 30 min at 37 °C in the dark. The fluorescence of the cells was measured using a Tecan microplate reader at an excitation wavelength of 485 nm and emission of 525 nm.

### JC-1 assay

The MES and MES-TP cells were cultured in 96-well plates at the concentration of 8000 cells/well, and then treated with 0.5 mg/ml ASP, 5 nM PTX, or the combination for 8 h. 20 uM JC-1 was added to each well and incubated for an additional 30 min at 37 °C. JC-1 production was detected by a Tecan plate reader at wavelengths of 535/590 nm and 485/535 nm.

### TMRE assay

Mitochondrial membrane potential was measured using the TMRE assay (Thermo Fisher Scientific), according to our previous protocol (Fan et al. 2022). The MES and MES-TP cells were cultured in 96-well black plates at 8000 cells/well for 24 h, and then treated with 0.5 mg/ml ASP, 5 nM PTX, or the combination for 8 h. The plates were then washed with PBS twice, and 100 ul PBS with 1 mM TMRE was added to each well. The plates were then incubated for 30 min at 37 °C. The fluorescence density in each well was detected using a microplate reader at an excitation of 548 nm and an emission of 573 nm.

### Adhesion assay

96-well plates were coated with 100 ul Laminin (12 ug/ml) at 4 °C overnight. The MES and MES-TP cells were seeded at 2.5 × 10^4^ per well and treated with 0.5 mg/ml ASP, 5 nM PTX, or the combination for 2 h. 100 ul of 5% glutaraldehyde was added to each well, and the plates were incubated for 30 min at room temperature. After a PBS wash, each well was stained with 100 ul of 0.1% purple crystal for 20 min. The absorption values were detected at 570 nm with a microplate reader (Tecan, Durham, NC).

### Wound healing assay

The MES and MES-TP cells were seeded in 6-well plates at a density of 4.5 × 10^5^/well for 24 h. A wound was created by scratching a line across the bottom of the plate using a 20-μL pipette tip. The cells were then treated with 0.5 mg/ml ASP, 5 nM PTX, or the combination for 48 h. Photos were taken at 24 h and 48 h after treatment. The distance between the scratch was calculated using ImageJ analysis software. This experiment was repeated three times for consistency.

### Western blotting

The MES and MES-TP cells were treated with 0.5 mg/ml ASP, 5 nM PTX, or the combination overnight, followed by total protein extraction with RIPA lysis buffer. The protein concentrations were measured with a BCA protein-assay kit (Thermo Scientific, Waltham, MA). Next, proteins were separated by 10–12% SDS-PAGE (Bio-Rad Laboratories, Hercules, CA) and transferred to PVDF membranes (Millipore, Billerica, MA). After blocking with 5% non-fat milk for 1 h at room temperature, the membranes were incubated with the primary antibodies overnight at 4 °C, and then incubated with the secondary antibodies for 1 h at room temperature. The PVDF membranes were visualized by Super Signal WestPico™ (Thermo Scientific) and analyzed using the Bio-Rad ChemiDoc™ image system (Hercules, CA).

### Statistical analysis

Experiments were performed in triplicate with at least three independent experiments unless indicated, and all data are presented as a mean ± the standard error of the mean. Statistical tests and graphs were generated by GraphPad Prism 8 software. Student’s *t*-test or one-way ANOVA was used for comparisons between groups. *p* < 0.05 was considered statistically significant.

## Results

### The combination of ASP and PTX exerted enhanced growth inhibition in OC cells

**Fig. 1:**
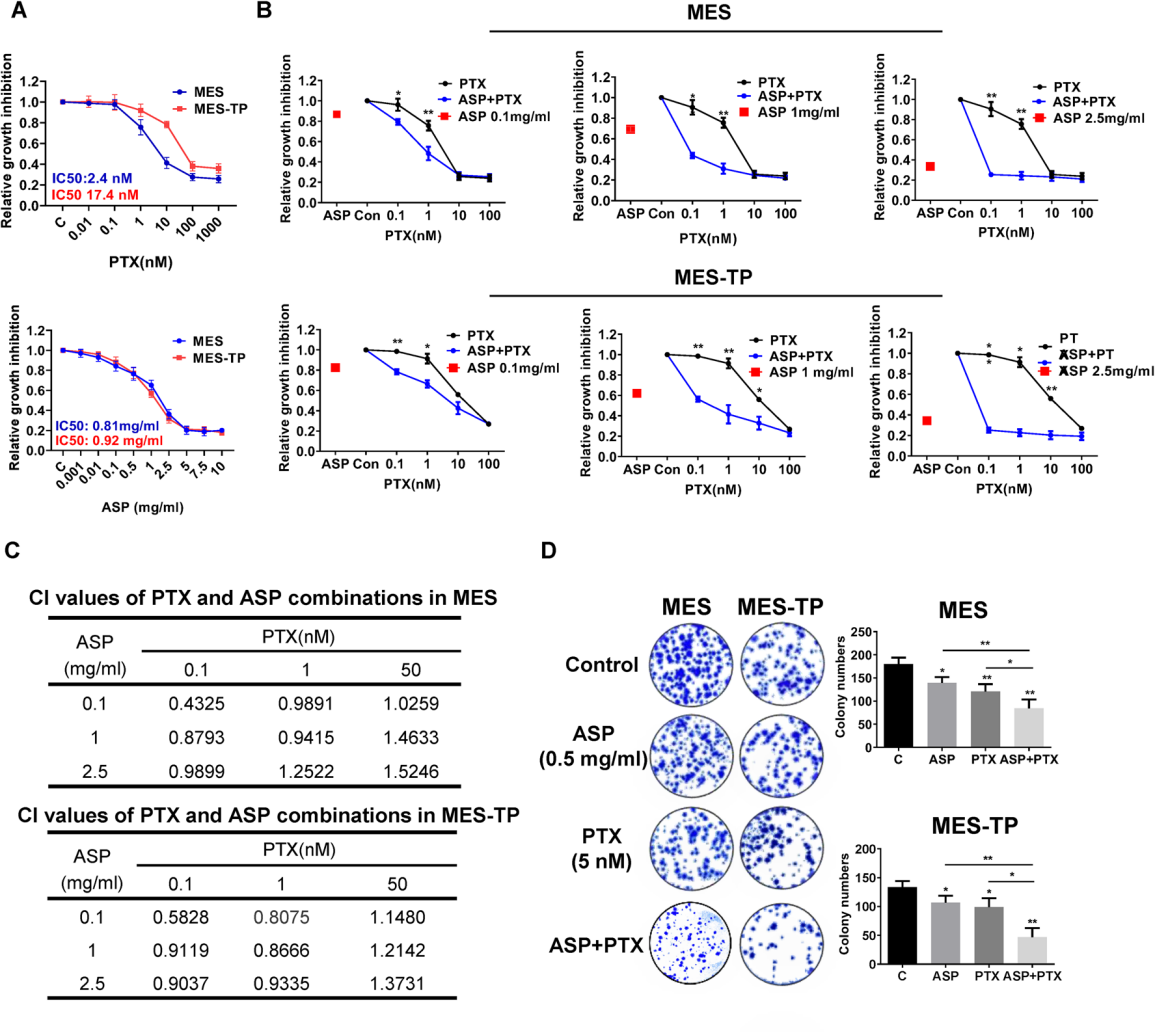
Effect of ASP, PTX or both in combinations on OC cell viability. The MES and MES-TP cells were seeded in 96-well plates at a density of 4000/well and treated with 0.5 mg/ml ASP, 5 nM PTX or the ASP/PTX combination at different doses for 72 h. Cell proliferation was measured by MTT assay. ASP inhibited cell proliferation in the MES and MES-TP cells (**A**). The combination of ASP and PTX at low doses showed synergistic inhibitory effects on cell proliferation in both cell lines (**B**). The combination index (CI) was calculated using the Bliss Independence model (**C**). CI < 1, synergistic effect; CI = 1, additive effect; CI > 1, antagonistic effect. The MES and MES-TP cells were treated with ASP (0.1 mg/ml), PTX (1 nM) and the combination treatment for 24 h, and then the cells were cultured for an additional 2 weeks, followed by colony assay assessment. The combination treatment produced more inhibitory effects on colony formation in both cell lines (**D**). *p < 0.05, **p < 0.01. The experiments were repeated three times

We first examined the inhibitory effect of ASP and PTX in the MES-TP cell lines as well as in the parental MES cells. Both cell lines were treated with different concentrations of ASP or PTX for 72 h. MTT assays showed that ASP had similar inhibitory effects on cell viability in both cell lines, with IC50s of 0.92 mg/ml in the MES-TP cells and 0.81 mg/ml in MES cells. PTX exerted significant cytotoxicity in MES cells with an IC50 of 2.4 nM as compared to a much higher IC50 of 17.42 nM in the MEX-TP cells (Fig. 1A). To evaluate the synergistic effect of the combination of PTX and ASP in MES and MES-TP cells, both cell lines were treated with different doses (approximating IC20, IC50 and IC70) of ASP alone, PTX alone, and the combination of the two agents for 72 h. The combination of 0.1 mg/ml or 0.5 mg/ml ASP and different doses of PTX was significantly more effective than PTX alone in inhibiting cell proliferation in the MES and MES-TP cells. ASP doses below the IC50 did not increase the sensitivity to PTX in both cells (Fig. 1B). Based on the cell viability at each combination point, we used the Bliss Independence model to calculate the combination Index values (CI) (Foucquier and Guedj 2015). The CI value was lower than 1.0 when both cells were treated with the combination of PTX and ASP at low doses, suggesting that the combination of ASP and PTX at low doses generated synergetic effects in growth inhibition in both the MES and MES-TP cells (Fig. 1C).

As clonogenic assays remain the gold standard for assessing cancer cell response to therapy, we evaluated the effects of ASP, PTX and the combination on colony formation in both cell lines. The MES and MES-TP cells were treated with 0.1 mg/ml ASP, 1 nM PTX or the combination for 24 h, and then cultured for another 2 weeks. ASP and PTX reduced colony formation in both cell lines; however, the combination treatment produced a more potent inhibition on colony formation (Fig. 1D). In the MES and MES-TP cells, 1 nM PTX reduced colony formation by 32.8% and 25.1%, respectively, while 0.1 mg/ml ASP decreased colony formation by 23.2% and 20.3%, respectively. The combination of ASP and PTX reduced colony formation by 53.1% in the MES cells and 64.8% in the MES-TP cells compared to control groups (*p* < 0.01). These results demonstrate that the combination of ASP and PTX exhibited effective toxicity not only in the PTX-sensitive MES cells, but also in the PTX-resistant MES-TP cell line.

### The combination of ASP and PTX exhibited more efficient induction of apoptosis in OC cells

**Fig. 2:**
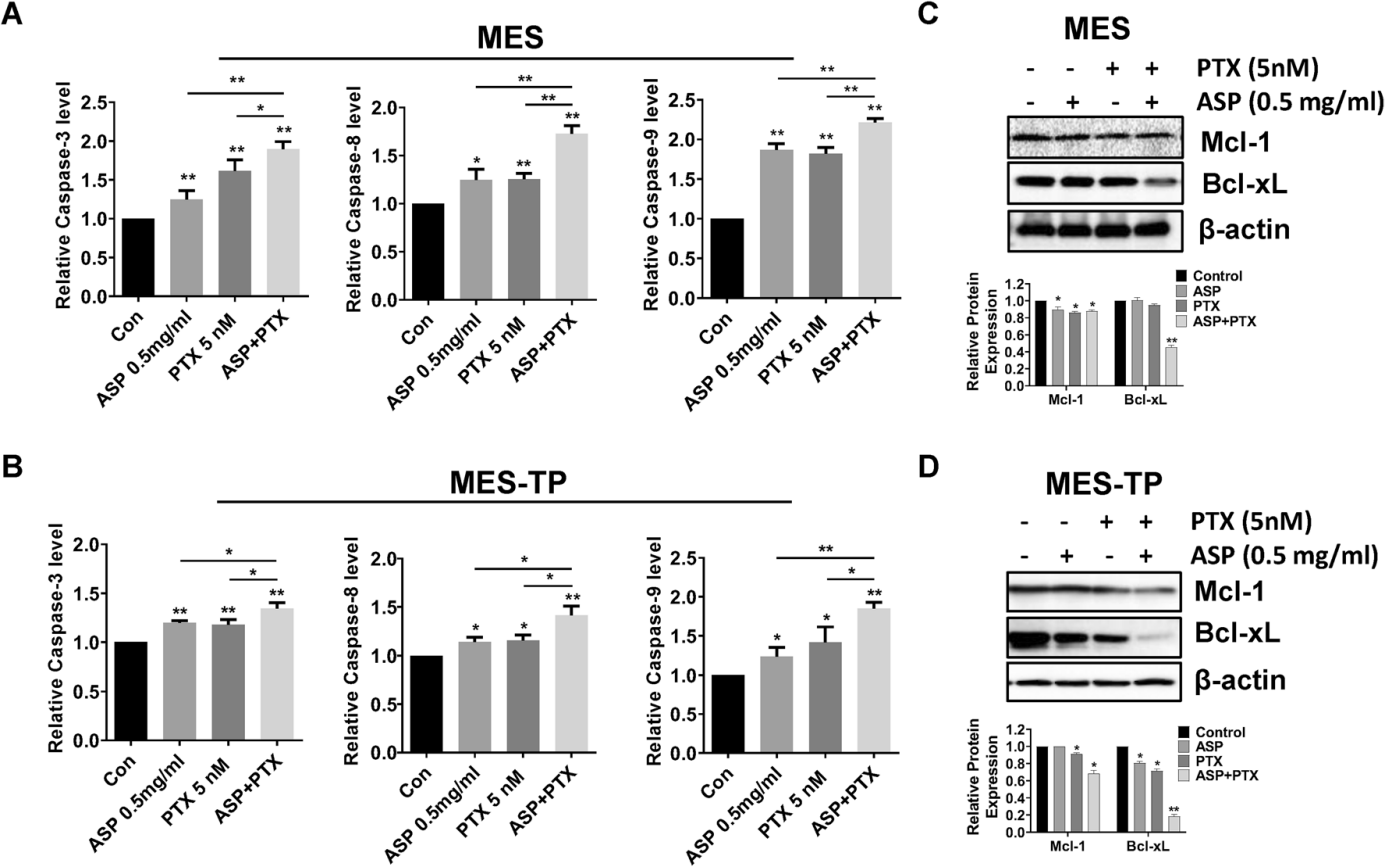
The effect of ASP, PTX or the combination on apoptosis. The MES and MES-TP cells were treated with ASP (0.5 mg/ml), PTX (5 nM) or the combination for 14–16 h. ELISA assay was used to detect cleaved caspase 3, 8 and 9 activities. ASP or PTX significantly increases the activity of caspase 3, 8 and 9, with the greatest effect on combination treatments in both cell lines (**A** and **B**). Both cell lines were treated with 0.5 mg/ml ASP, 5 nM PTX and the combination for 24 h, and the expression of MCL-1 and BCL-XL was assessed by western blotting. PTX significantly decreased the expression of MCL-1 and BCL-XL in both cell lines, the combination treatment produced more inhibitory effects on MCL-1 and BCL-XL compared to ASP or PTX alone in both cell lines (**C** and **D**). *p < 0.05, **p < 0.01. The experiments were repeated at least 2 times

ASP and PTX are both known to induce apoptosis in cancer cells. To evaluate the effect of ASP, PTX or the combination of ASP and PTX on apoptosis, we used ELISA assays to detect the changes of caspase 3, 8 and 9 in the OC cells. Increased activity of cleaved caspase 3, cleaved caspase 8, and cleaved caspase 9 was observed in the MES and MES-TP cells after 14–16 h of exposure to 0.5 mg/ml ASP, 5 nM PTX and the combination treatment (p < 0.01). Both cell lines had similar responses to apoptosis induction in response to these treatments. The combination treatments had more potential to highly induce activity of cleaved caspase 3, 8 and 9 compared to ASP alone and PTX alone in both cell lines (Fig. 2A and B, *p* < 0.05). Furthermore, western blotting analysis showed that 0.5 mg/ml ASP decreased the expression of MCL-1 in the MES cells and BCL-XL expression in the MES-TP cells, and 5 nM PTX reduced MCL-1 and BCL-XL expression in both cell lines after 24 h of treatment. Similarly, the combination treatment produced potent inhibition of MCL-1 and BCL-XL expression compared to ASP or PTX alone in both cell lines (Fig. 2C and D).

### The combination of ASP and PTX displayed effective induction of cellular stress in OC cells

**Fig. 3:**
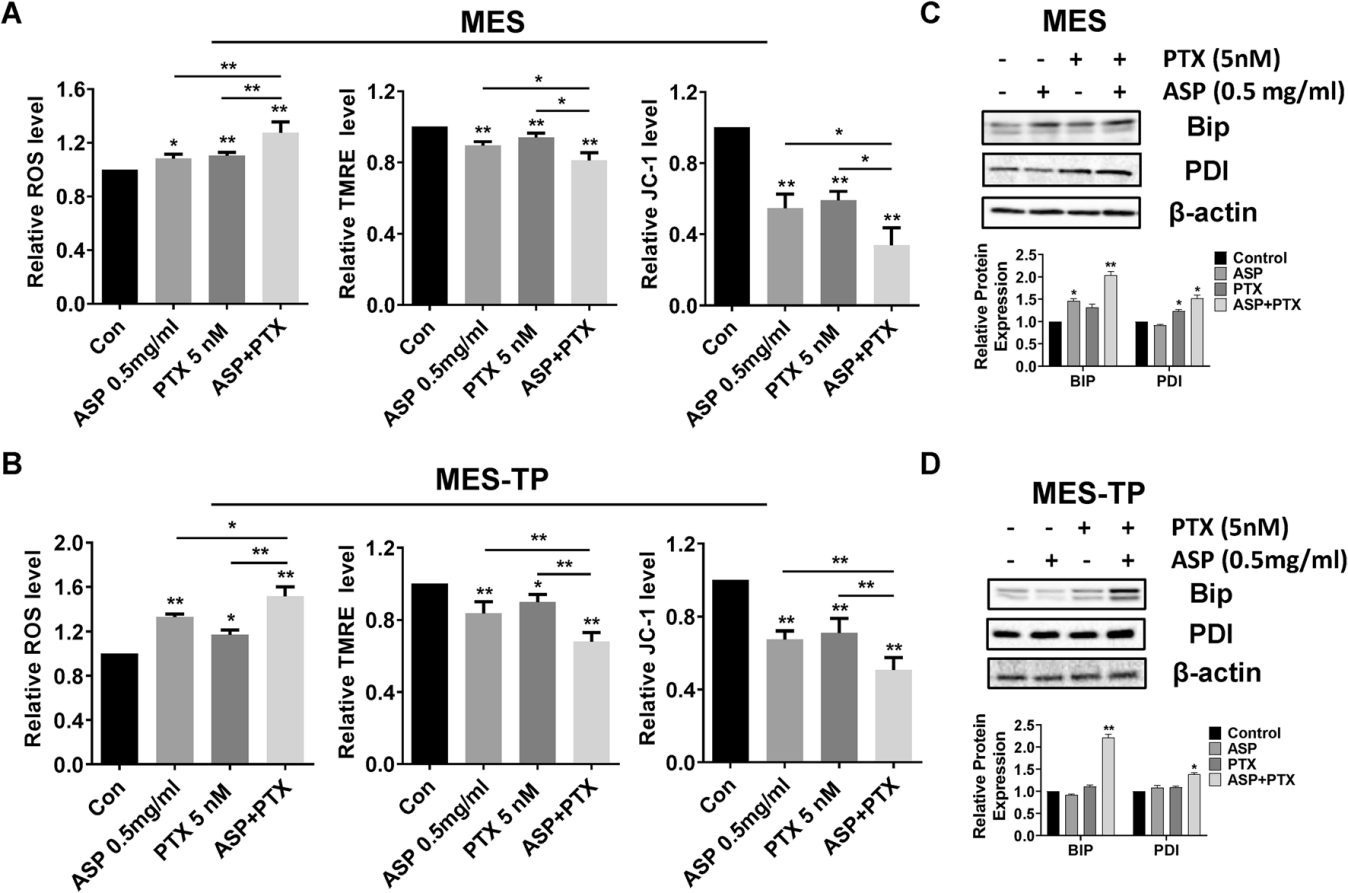
The effect of ASP, PTX or the combination on cellular stress. The MES and MES-TP cells were treated with ASP (0.5 mg/ml), PTX (5 nM) or the combination for 8 h. Intracellular ROS production was determined by DCFDA assay. Mitochondrial membrane potential was detected by JC-1 assay and TMRE assay. ASP, PTX and the combination treatment increased cellular ROS levels and reduced mitochondrial membrane potential, with the greatest effect on combination treatments in both cell lines (**A** and **B**). Both cell lines were treated with 0.5 mg/ml ASP, 5 nM PTX or the combination at the indicated doses for 24 h. Western blotting was performed to assess protein expression levels of the cellular stress markers, PDI and Bip (**C** and **D**). The combination of ASP and PTX resulted in more increased PDI and Bip expression compared to control and single agent groups for both cell lines. *p < 0.05, **p < 0.01. The experiments were repeated at least 3 times

To define the combined effects of ASP and PTX on oxidative stress, DCFH-DA assay was used to detect cellular ROS production. Treatment of the MES and MES-TP cells with ASP, PTX or the combination for 8 h increased intracellular ROS levels, and this increase was more pronounced with the combination treatment. Combination treatment resulted in a 32.2% increase in the MES cells and a 54.6% in the MES-TP cells compared to the control cells (Fig. 3A, *p* < 0.01). We next examined the combined effects of ASP and PTX on mitochondrial membrane potential (ΔΨm). MES cells treated with 0.5 mg/ml ASP, 5 nM PTX, or the combination significantly reduced ΔΨm, with the most pronounced reduction in the combination group compared to ASP or PTX alone (*p* < 0.01). Similarly, a significantly superior reduction in ΔΨm occurred in the MES-TP cells with the same treatments (Fig. 3A and B). Western blotting results showed ASP combined with PTX significantly increased PDI and Bip expression compared to control and single agent groups for both cell lines (Fig. 3C and D).

### The combined effects of ASP and PTX on invasion in OC cells

**Fig. 4:**
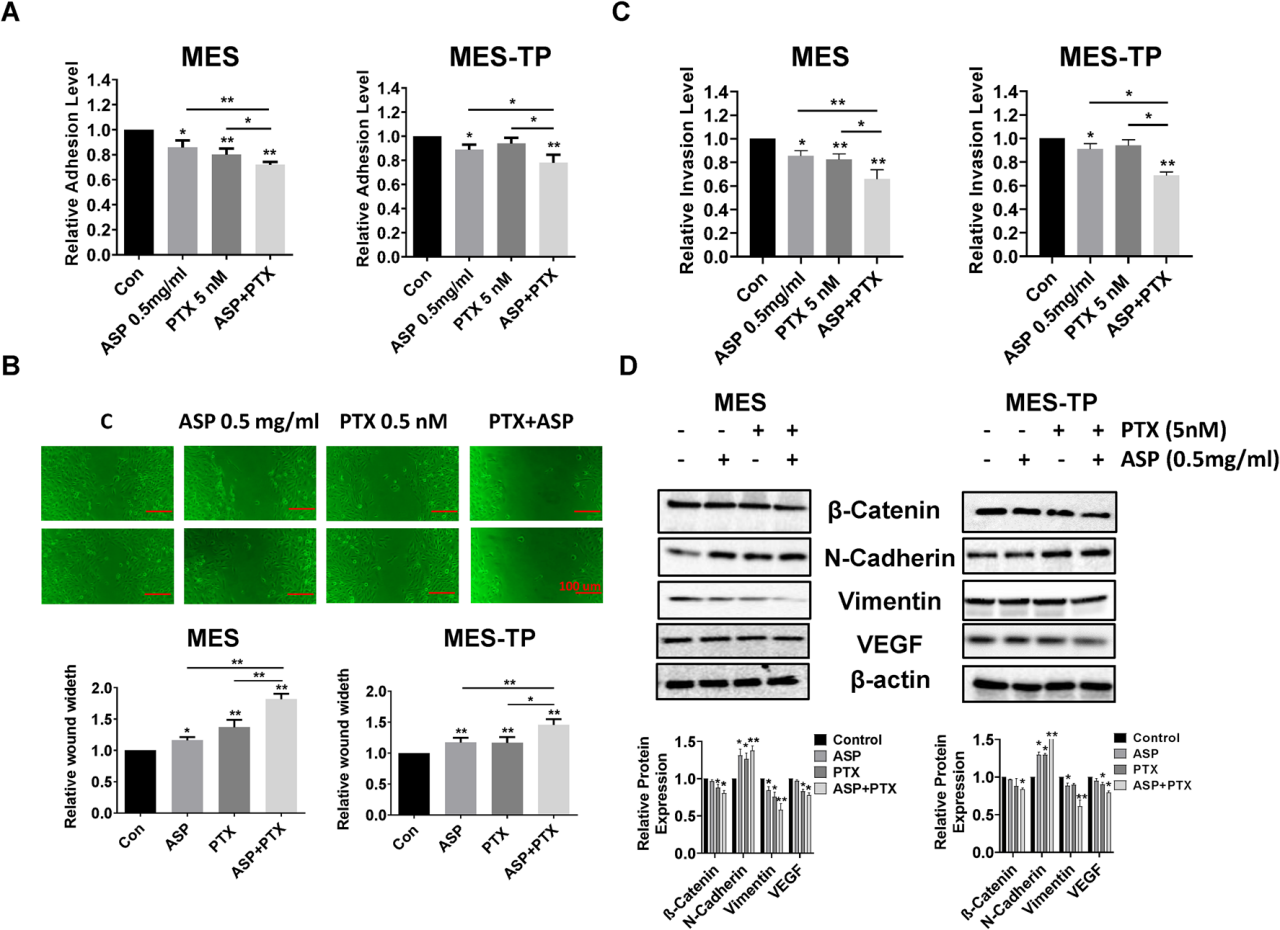
The effect of ASP, PTX or the combination on cell adhesion and invasion. The MES and MES-TP cells were treated with ASP (0.5 mg/ml), PTX (5 nM) or the combination for 4 h. Cell adhesion was determined by laminin adhesion assay (**A**). Transwell assay was used to assess effects on invasion in both cell lines (**B**). Both cell lines were treated with ASP (0.5 mg/ml), PTX (5 nM) and the combination for 48 h. Wound healing assay was used to evaluate migration/invasive ability (**C**). ASP and PTX significantly reduced the adhesion, migratory and invasive abilities of both cell lines, while the combination treatments exhibited more inhibitory effects. The protein expression of EMT biomarkers was determined by Western blot analysis after 24 h of treatment in both cells (**D**). Scale bar: 100 um. The experiments were repeated at least 3 times. *p < 0.05, **p < 0.01

To investigate the combined effects of ASP and PTX on cell migration and invasion, we performed laminin-1 adhesion, wound healing and transwell assays in the MES and MES-TP cells. Inhibition of adhesion was observed in ASP-treated and PTX-treated MES and ASP-treated MES-TP cells. 5 nM PTX alone did not reduce cellular adhesion in the MES-TP cells. Importantly, the combination of 0.5 mg/ml ASP with 5 nM PTX significantly reduced cell adhesion in both cells compared to controls as well as ASP or PTX alone (Fig. 4A, *p* < 0.05). A similar phenomenon was observed in both cell lines when we used the transwell assay to detect cell invasion. 0.5 mg/ml ASP inhibited cellular invasion in both cell lines while 5 nM PTX reduced cellular invasion in the MES cells but not in the MES-TP cells. Cellular invasion was synergistically inhibited in the presence of the combination of ASP and PTX (Fig. 4B, *p* < 0.05). Results from the wound healing assay showed that 0.5 mg/ml ASP and 5 nM PTX inhibited the motility of MES cells. In MES-TP cells, ASP displayed inhibitory effect on cell motility but PTX alone did not affect cell motility. The combination of 0.5 mg/ml ASP and 5 nM PTX had a greater ability to inhibit cellular migration in both cell lines compared to vehicle, ASP or PTX alone (Fig. 4C, *p* < 0.05).

Due to the role of epithelial mesenchymal transition (EMT) in the process of cell invasion, we used western blotting to examine changes in EMT markers with ASP/PTX treatment. The combination of ASP and PTX caused considerable up-regulation of N-cadherin and downregulation of vimentin, VEGF and B-catenin in both cell lines after 24 h of treatment (Fig. 4D). These results confirm that ASP combined with PTX effectively suppressed both migration and invasion in PTX-sensitive or -resistant OC cell lines.

### The combined effects of ASP and PTX on the AMPK and AKT/S6 pathways in OC cells

**Fig. 5:**
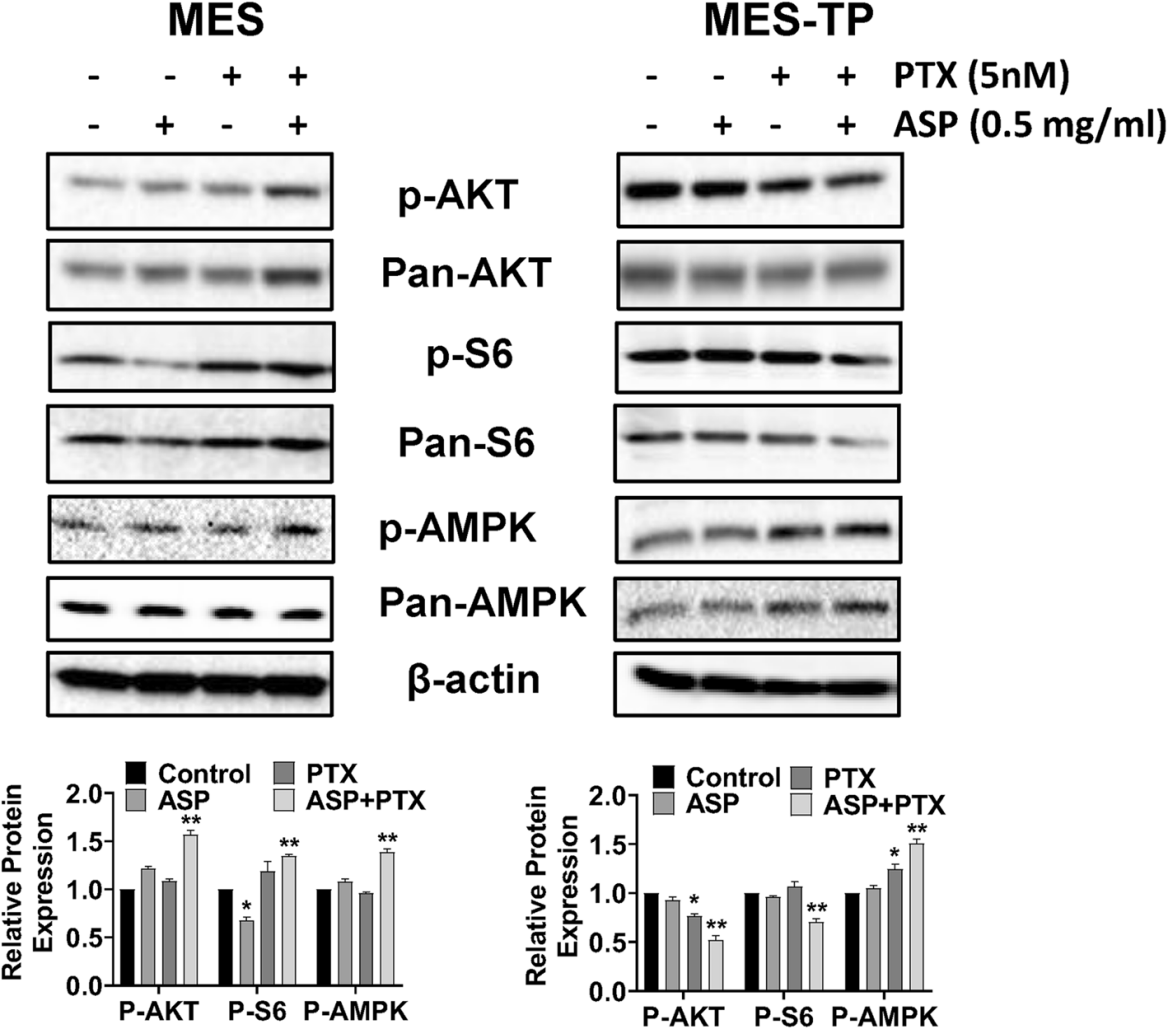
The effect of ASP, PTX or the combination on the AKT/mTOR/S6 and AMPK pathways. The MES and MES-TP cells were treated with ASP (1 mg/ml), PTX (5 nM) or the combination for 24 h. Western blotting was used to determine the expression of phosphorylated AKT and S6 after drug treatment. The results showed that the combination of ASP and PTX activated AMPK phosphorylation in both cell lines. In the MES cells, combination treatment resulted in increased expression of phosphorylated AKT and S6. In the MES-TP cells, the combination treatment decreased the expression of phosphorylated AKT and S6. In addition, the combination treatment significantly increased the expression of phosphorylation of AMPK in both cell lines. *p < 0.05, **p < 0.01. The experiments were repeated at least 3 times

The AKT/mTOR pathway is a major pathway involved in carcinogenesis, progression, and drug resistance in OC (Guo et al. 2018). AMPK signaling controls energy balance, cell proliferation and survival in cancer (Russell and Hardie 2020). To evaluate whether the AKT/mTOR and AMPK pathways are responsible for the inhibitory effect of ASP and PTX on MES and MES-TP cells, both cell lines were treated with 0.5 mg/ml ASP, 5 nM PTX and the combination of ASP and PTX for 24 h. AKT/mTOR activity was determined by phosphorylation of AKT (ser473) and S6 (Ser235/236), and AMPK activation was evaluated by AMPK phosphorylation on Thr172. PTX slightly increased phosphorylated AMPK expression in the MES-TP cells. 0.5 mg/ml ASP combined with 5 nM PTX was more effective in increasing the expression of AMPK phosphorylation in both cell lines. In the MES cells, PTX increased the expression of S6 phosphorylation, whereas ASP decreased the expression of S6 phosphorylation compared to the vehicle control. Combination treatment resulted in a significant increase in phosphorylation of AKT and S6. In the MES-TP cells, PTX, but not ASP, reduced AKT phosphorylation, and ASP and PTX single agent treatments did not change the expression of phosphorylation of S6. Combination treatment further reduced the expression of AKT and S6 phosphorylation (Fig. 5). Taken together, these data suggest that activation of the AMPK pathway is one of the mechanisms responsible for combination therapy-mediated cell growth inhibition, and that the AKT/mTOR/S6 pathway exhibits distinct roles in this process in PTX-sensitive and -resistant cell lines.

### The combined effects of ASP and PTX on DNA damage pathways in OC cells

**Fig. 6:**
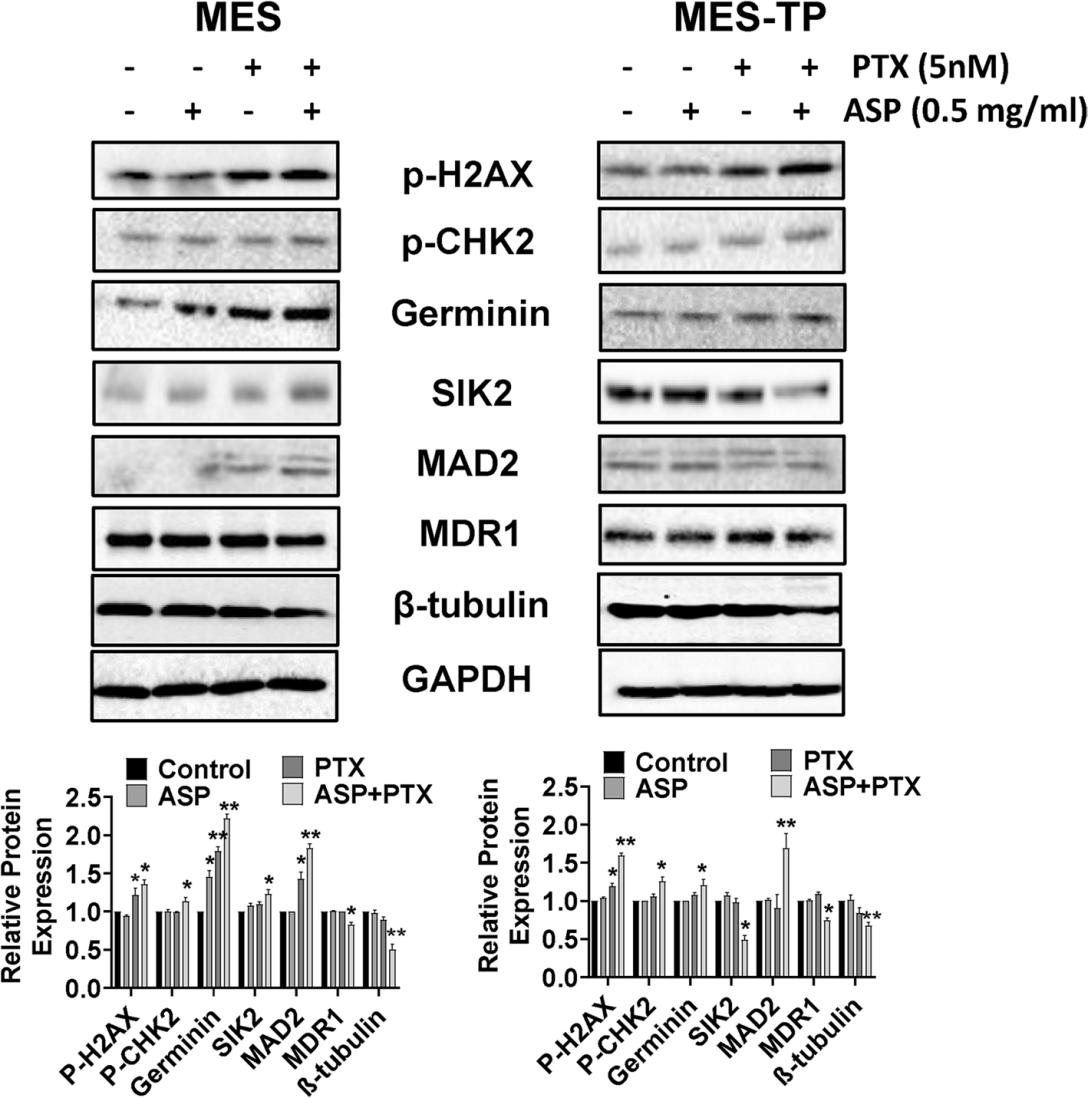
The effect of ASP, PTX or the combination on DNA damage pathways. The MES and MES-TP cells were treated with ASP (0.5 mg/ml), PTX (5 nM) or the combination for 24 h. The DNA damage markers p-H2AX, p-CHK2 and geminin were detected by western blotting. The combination treatment increased the expression of p-H2AX, p-CHK2 in both cell lines. In addition, the combination treatment resulted in decreased expression of MDR1 and β-tubulin in both cell lines, increased expression of SIK1 and MAD2 in MES cells, and reduced expression of SIK1 and MAD2 in MES-TP cells. *p < 0.05, **p < 0.01. The experiment were repeated at least 3 times

PTX resistance involves modulating DNA repair, multi-drug resistance (MDR) expression, and microtubule changes in cancer. To explore the combined effects of ASP and PTX on DNA damage pathways in MES and MES-TP cell lines were treated with 0.5 mg/ml ASP, 5 nM PTX or the combination for 24 h, and western blotting was performed. PTX treatment was capable of increasing the expression of DNA damage markers, geminin and phosphorylation of γ-H2AX, in the MES and MES-TP cells, whereas ASP did not change the expression of γ-H2AX or CHK2 phosphorylation. The combination of ASP and PTX exhibited stronger effects in inducing H2AX and CHK2 phosphorylation and increasing the expression of geminin in both cells (Fig. 6), suggesting that the synergistic growth inhibition induced by combination treatment was dependent on DNA damage pathways.

Since the degree of MDR1 expression strongly correlated with resistance to PTX in cancer cells (Mechetner et al. 1998), we assessed whether combined effects of ASP and PTX had any relevant role in MDR1 expression by western blotting after treatment with of ASP, PTX or and the combination in both cell lines for 24 h. 0.5 mg/ml ASP or 5 nM PTX alone did not change the expression of MDR1 in the MES cells; however, PTX increased MDR1 expression in the MES-TP cells. ASP combined with PTX reduced MDR1 expression in the MES cells and blocked the PTX-induced MDR increase in the MES-TP cells.

Given that microtubule-associated proteins (MAPs) are involved in regulating microtubule dynamics, stability of microtubules and PTX sensitivity (Safinya et al. 2016; Shi and Sun 2017), the expression of MAPs related proteins, Salt Inducible Kinase 2 (SIK2), mitotic arrest deficient 2 (MAD2) and β-tubulin, was detected by western blotting after treatment with ASP, PTX or the combination for 24 h. Highly expressed SIK2 and MAD2 effectively induces PTX resistance in ovarian and breast cancer (Ahmed et al. 2010; Bargiela-Iparraguirre et al. 2014). 0.5 mg/ml ASP treatment alone did not affect the expression SIK2, MAD2 or β-tubulin in either cell line. 5 nM PTX slightly increased MAD2 in the MES cells, but decreased MAD2 in the MER-TP cells. PTX also marginally decreased the expression of β-tubulin in both cell lines, whereas the combination treatment significantly reduced its expression. In addition, the combination treatment led to increased expression of SIK2 and MAD2 in the MES cells, but conversely decreased SIK2 and MAD2 in the MES-TP cells compared to the control, ASP and PTX groups. These results indicate that combination treatment elicited different effects on MAPs in PTX-sensitive and -resistant OC cells.

## Discussion

The resistance of ovarian tumor cells to PTX is a key factor in limiting the effective treatment of advanced OC with PTX. Therefore, the development of safe and effective adjunct therapies to overcome PTX resistance is critical to improving the survival rate of patients with advanced or recurrent OC. In the present study, we assessed the therapeutic potential of the combination of ASP and PTX on PTX-sensitive and -resistant OC cells by investigating the anti-tumorigenic effects of ASP alone, PTX alone and the combination treatment against both cell lines. Our results demonstrated that combined ASP and PTX at low concentrations was highly synergistic in the inhibition of cell proliferation, migration and invasion, and more effectively induced apoptosis and cellular stress in MES and MES-TP cells. These synergistic responses appear to result from different underlying effects on the AKT/mTOR signaling pathway and DNA damage pathways in PTX-sensitive MES cells and PTX-resistant MES-TP cells.

Although natural products of plant origin have traditionally been considered complementary nutritional supplements, there is a long history of evidence suggesting that certain natural products or natural plant active ingredients have anti-cancer effects. Now more than 60% of current anti-cancer drugs are derived from natural sources in various ways (Lin et al. 2020; Cragg and Pezzuto 2016). ASP is a popular healthy vegetable that is rich in steroidal saponins, saccharides, flavonoids, phenolic compounds, among others. These phytochemicals from ASP exhibit broad anti-tumor activities in different cancer cells, including inhibition of cell proliferation, induction of apoptosis and cell cycle arrest, and inhibition of invasion in vitro and in vivo (Romani et al. 2021; Zhang et al. 2018b, 2020, 2021a; Xiang et al. 2014; Wang et al. 2013). Early studies found that asparagus polysaccharide effectively increased sensitivity to mitomycin in hepatocellular carcinoma cells and mouse models (Xiang et al. 2014). Our previous results confirmed that ASP significantly inhibited cell viability and increased sensitivity to PTX in OC cells (Xu et al. 2021b). Given the cytotoxic effects of ASP and PTX, we selected three concentrations of ASP and PTX for synergistic studies in our current study, based on their IC50 values in MES and MES-TP cells. Low-dose ASP produced synergistic growth inhibitory effects and induced significant apoptosis and cellular stress responses, but only when combined with low-dose PTX as opposed to high-dose PTX. In addition, high-dose ASP did not elicit a synergistic response when combined with different concentrations of PTX in either cell line. These results suggest that ASP has distinctly different mechanisms of inhibiting cell growth and inducing apoptosis in MES and MES-TP cells compared to PTX. Furthermore, increased sensitivity of tumor cells to low-dose PTX via ASP is clinically relevant, especially in PTX-resistant OC.

The mechanisms responsible for PTX resistance are multifactorial and included undesired DNA repair, up-regulation of anti-apoptotic proteins and efflux pump activity, increased tubulin isoforms, activation of pro-survival pathways, enhanced function of drug-metabolizing enzymes, among others (Mosca et al. 2021; Tendulkar and Dodamani 2021; Guo et al. 2018; Maloney et al. 2020). Recent studies have shown that most natural compounds reduce chemoresistance by inhibiting the expression of the multi-drug resistance gene (MDR) or reducing MDR protein activity in cancer cells (Turrini et al. 2014; Vaidyanathan et al. 2016; Yan et al. 2020). Since MES-TP cells are resistant to PTX, MES-TP cells expressed higher MDR1 than MES cells. ASP and PTX did not increase or decrease MDR1 expression in MES cells; however, in MES-TP cells, PTX-induced MDR1 overexpression. The combination of ASP and PTX reduced MDR1 expression in both cells. These results indicate that ASP may be specifically effective in reversing PTX-induced resistance in OC cells. This is the first demonstration that ASP reduces PTX-induced MDR1 expression in PTX-resistant OC cells.

Elevated intracellular ROS production is well known to cause oxidative DNA damage that triggers the activation of apoptosis through the extrinsic and intrinsic apoptotic pathways in cancer cells (Mohiuddin and Kasahara 2021; Srinivas et al. 2019). PTX increases the level of ROS in many types of cancer cells, including PTX-resistant cell lines (Mohiuddin and Kasahara 2021; Sugiyama et al. 2020; Chen et al. 2017). Phosphorylated γ-H2AX and CHK2 as sensitive indicators of DNA damage are commonly used to assess PTX-induced DNA damage or DNA replication stress (Kimani et al. 2021; Gutiérrez-González et al. 2013). In addition, altered microtubule dynamics, due to changes in the expression or post-translational modifications of microtubule-associated proteins (MAPs), may contribute to tumor resistance to PTX in a wide range of cancer types (Safinya et al. 2016; Orr et al. 2003; Xie et al. 2016). In the current study, PTX increased the expression of geminin and phosphorylated γ-H2AX in the MES and MES-TP cells. The combination of ASP and PTX exhibited more potent effects on geminin, phosphorylated γ-H2AX and CHK2 in both cells. Similar results occurred with β-tubulin changes following ASP and PTX treatment. However, we observed that PTX and the combination therapy had opposite effects on the expression of the MAP proteins SIK2 and MAD2 and activity of the AKT/mTOR/S6 pathways in MES versus MES-TP cell lines. These results provide evidence that the combination of ASP and PTX synergically inhibited cell proliferation via DNA damage pathways and suppressed microtubule dynamics in both OC cell lines, and the MARs and AKT/mTOR pathway mediated the inhibitory effect through different mechanisms in PTX-sensitive and -resistant OC cells.

PTX-induced epithelial-to-mesenchymal transition (EMT) involves several different dysregulated pathways involved in regulating proliferation, apoptosis, and conventional EMT (Jia et al. 2012; Kajiyama et al. 2007). Accumulating studies show that the EMT process involves not only tumor invasion and metastasis but also PTX resistance in cancer cells (Yang et al. 2014; Ashrafizadeh et al. 2021). Microtubule stabilization by PTX significantly reduces cancer cell invasion through inhibition of cytoskeletal network remodeling in invadopodia maturation (Hwang et al. 2019). ASP has been shown to inhibit cell motility and invasion of breast cancer via modulating the Rho GTPase signaling pathway (Wang et al. 2013). The asparanin A form of ASP has been shown to reduce migration and invasion via the Ras/ERK/MAPK pathway in endometrial cancer cells and mouse models (Zhang et al. 2021b). We recently demonstrated that inhibition of invasion by ASP may be related to ASP’s effects on the regulation of EMT and angiogenesis in OC in vitro and in vivo (Xu et al. 2021a). In this study, ASP combined with PTX significantly decreased adhesion and invasion through EMT process compared to ASP alone, PTX alone and vehicle control in MES and MES-TP cells. Although more evidence is needed to better understand the potential anti-metastatic mechanism of this combination, these results support further investigation of the combination of ASP and PTX treatment in mouse models of OC.

## Conclusions

Our data demonstrate that the combination of ASP and PTX synergistically inhibited cell viability, induced apoptosis and reduced invasion in PTX-sensitive and -resistant OC cell lines compared to treatment with either drug alone. These findings have potential clinical implications for the development of more effective OC treatment strategies, especially in platinum-resistant advanced and relapsed OC, a long-standing clinical dilemma for this highly lethal disease.

## Supplementary Information

Below is the link to the electronic supplementary material.

Supplementary file1 (DOCX 37 KB)

Supplementary file2 (PDF 6993 KB)

## Data Availability

The raw data supporting the conclusions of this article will be made available by the authors, without undue reservation.

## References

[CR1] Acquaviva R, Malfa GA, Loizzo MR, Xiao J, Bianchi S, Tundis R (2022) Advances on natural abietane, labdane and clerodane diterpenes as anti-cancer agents: sources and mechanisms of action. Molecules. 10.3390/molecules27154791 35897965 10.3390/molecules27154791 PMC9330018

[CR2] Ahmed AA, Lu Z, Jennings NB, Etemadmoghadam D, Capalbo L, Jacamo RO, Barbosa-Morais N, Le XF, Vivas-Mejia P, Lopez-Berestein G, Grandjean G, Bartholomeusz G, Liao W, Andreeff M, Bowtell D, Glover DM, Sood AK, Bast RC Jr (2010) SIK2 is a centrosome kinase required for bipolar mitotic spindle formation that provides a potential target for therapy in ovarian cancer. Cancer Cell 18(2):109–121. 10.1016/j.ccr.2010.06.018 20708153 10.1016/j.ccr.2010.06.018 PMC3954541

[CR3] Ashrafizadeh M, Mirzaei S, Hashemi F, Zarrabi A, Zabolian A, Saleki H, Sharifzadeh SO, Soleymani L, Daneshi S, Hushmandi K, Khan H, Kumar AP, Aref AR, Samarghandian S (2021) New insight towards development of paclitaxel and docetaxel resistance in cancer cells: EMT as a novel molecular mechanism and therapeutic possibilities. Biomedicine & Pharmacotherapy = Biomedecine & Pharmacotherapie 141:111824 34175815 10.1016/j.biopha.2021.111824

[CR4] Bargiela-Iparraguirre J, Prado-Marchal L, Pajuelo-Lozano N, Jiménez B, Perona R, Sánchez-Pérez I (2014) Mad2 and BubR1 modulates tumourigenesis and paclitaxel response in MKN45 gastric cancer cells. Cell Cycle 13(22):3590–3601. 10.4161/15384101.2014.962952 25483095 10.4161/15384101.2014.962952 PMC4615044

[CR5] Chen FQ, Zhang JM, Fang XF, Yu H, Liu YL, Li H, Wang YT, Chen MW (2017) Reversal of paclitaxel resistance in human ovarian cancer cells with redox-responsive micelles consisting of α-tocopheryl succinate-based polyphosphoester copolymers. Acta Pharmacol Sin 38(6):859–873. 10.1038/aps.2016.150 28260803 10.1038/aps.2016.150 PMC5520180

[CR6] Cragg GM, Pezzuto JM (2016) Natural products as a vital source for the discovery of cancer chemotherapeutic and chemopreventive agents. Med Princ Pract 25(Suppl 2):41–59. 10.1159/000443404 26679767 10.1159/000443404 PMC5588531

[CR7] Das T, Anand U, Pandey SK, Ashby CR Jr, Assaraf YG, Chen ZS, Dey A (2021) Therapeutic strategies to overcome taxane resistance in cancer. Drug Resist Updat 55:100754. 10.1016/j.drup.2021.100754 33691261 10.1016/j.drup.2021.100754

[CR8] Fan Y, Wang J, Fang Z, Pierce SR, West L, Staley A, Tucker K, Yin Y, Sun W, Kong W, Prabhu V, Allen JE, Zhou C, Bae-Jump VL (2022) Anti-tumor and anti-invasive effects of ONC201 on ovarian cancer cells and a transgenic mouse model of serous ovarian cancer. Front Oncol 12:789450. 10.3389/fonc.2022.789450 35372029 10.3389/fonc.2022.789450 PMC8970020

[CR9] Foucquier J, Guedj M (2015) Analysis of drug combinations: current methodological landscape. Pharmacol Res Perspect 3(3):e00149. 10.1002/prp2.149 26171228 10.1002/prp2.149 PMC4492765

[CR10] Guo F, Zhang H, Jia Z, Cui M, Tian J (2018) Chemoresistance and targeting of growth factors/cytokines signalling pathways: towards the development of effective therapeutic strategy for endometrial cancer. Am J Cancer Res 8(7):1317–1331 30094104 PMC6079151

[CR11] Gutiérrez-González A, Belda-Iniesta C, Bargiela-Iparraguirre J, Dominguez G, García Alfonso P, Perona R, Sanchez-Perez I (2013) Targeting Chk2 improves gastric cancer chemotherapy by impairing DNA damage repair. Apoptosis 18(3):347–360 23271172 10.1007/s10495-012-0794-2

[CR12] Hoskins P, Vergote I, Cervantes A, Tu D, Stuart G, Zola P, Poveda A, Provencher D, Katsaros D, Ojeda B, Ghatage P, Grimshaw R, Casado A, Elit L, Mendiola C, Sugimoto A, D’Hondt V, Oza A, Germa JR, Roy M, Brotto L, Chen D, Eisenhauer EA (2010) Advanced ovarian cancer: phase III randomized study of sequential cisplatin-topotecan and carboplatin-paclitaxel vs carboplatin-paclitaxel. J Natl Cancer Inst 102(20):1547–1556. 10.1093/jnci/djq362 20937992 10.1093/jnci/djq362

[CR13] Hwang HJ, Oh MS, Lee DW, Kuh HJ (2019) Multiplex quantitative analysis of stroma-mediated cancer cell invasion, matrix remodeling, and drug response in a 3D co-culture model of pancreatic tumor spheroids and stellate cells. J Exp Clin Cancer Res 38(1):019–1225 10.1186/s13046-019-1225-9 PMC6567511 31200779

[CR14] Jayson GC, Kohn EC, Kitchener HC, Ledermann JA (2014) Ovarian cancer. Lancet 384(9951):1376–1388. 10.1016/s0140-6736(13)62146-7 24767708 10.1016/S0140-6736(13)62146-7

[CR15] Jia L, Zhang S, Ye Y, Li X, Mercado-Uribe I, Bast RC Jr, Liu J (2012) Paclitaxel inhibits ovarian tumor growth by inducing epithelial cancer cells to benign fibroblast-like cells. Cancer Lett 326(2):176–182 22902993 10.1016/j.canlet.2012.08.004 PMC3495569

[CR16] Kajiyama H, Shibata K, Terauchi M, Yamashita M, Ino K, Nawa A, Kikkawa F (2007) Chemoresistance to paclitaxel induces epithelial-mesenchymal transition and enhances metastatic potential for epithelial ovarian carcinoma cells. Int J Oncol 31(2):277–283 17611683

[CR17] Kimani S, Chakraborty S, Irene I, de la Mare J, Edkins A, du Toit A, Loos B, Blanckenberg A, Van Niekerk A, Costa-Lotufo LV, ArulJothi KN, Mapolie S, Prince S (2021) The palladacycle, BTC2, exhibits anti-breast cancer and breast cancer stem cell activity. Biochem Pharmacol 190(114598):9 10.1016/j.bcp.2021.114598 33979647

[CR18] Lin SR, Chang CH, Hsu CF, Tsai MJ, Cheng H, Leong MK, Sung PJ, Chen JC, Weng CF (2020) Natural compounds as potential adjuvants to cancer therapy: preclinical evidence. Br J Pharmacol 177(6):1409–1423. 10.1111/bph.14816 31368509 10.1111/bph.14816 PMC7056458

[CR19] Maloney SM, Hoover CA, Morejon-Lasso LV, Prosperi JR (2020) Mechanisms of taxane resistance. Cancers 12(11):3323 33182737 10.3390/cancers12113323 PMC7697134

[CR20] Mechetner E, Kyshtoobayeva A, Zonis S, Kim H, Stroup R, Garcia R, Parker RJ, Fruehauf JP (1998) Levels of multidrug resistance (MDR1) P-glycoprotein expression by human breast cancer correlate with in vitro resistance to taxol and doxorubicin. Clin Cancer Res 4(2):389–398 9516927

[CR21] Mohiuddin M, Kasahara K (2021) Paclitaxel impedes EGFR-mutated PC9 cell growth via reactive oxygen species-mediated DNA damage and EGFR/PI3K/AKT/mTOR signaling pathway suppression. Cancer Genomics Proteomics 18(5):645–659 34479917 10.21873/cgp.20287 PMC8441765

[CR22] Moisan F, Francisco EB, Brozovic A, Duran GE, Wang YC, Chaturvedi S, Seetharam S, Snyder LA, Doshi P, Sikic BI (2014) Enhancement of paclitaxel and carboplatin therapies by CCL2 blockade in ovarian cancers. Mol Oncol 8(7):1231–1239. 10.1016/j.molonc.2014.03.016 24816187 10.1016/j.molonc.2014.03.016 PMC4801026

[CR23] Mosca L, Ilari A, Fazi F, Assaraf YG, Colotti G (2021) Taxanes in cancer treatment: activity, chemoresistance and its overcoming. Drug Resist Updat 54:100742. 10.1016/j.drup.2020.100742 33429249 10.1016/j.drup.2020.100742

[CR24] Muhammad N, Usmani D, Tarique M, Naz H, Ashraf M, Raliya R, Tabrez S, Zughaibi TA, Alsaieedi A, Hakeem IJ, Suhail M (2022) The role of natural products and their multitargeted approach to treat solid cancer. Cells. 10.3390/cells11142209 35883653 10.3390/cells11142209 PMC9318484

[CR25] Orr GA, Verdier-Pinard P, McDaid H, Horwitz SB (2003) Mechanisms of Taxol resistance related to microtubules. Oncogene 22(47):7280–7295 14576838 10.1038/sj.onc.1206934 PMC4039039

[CR26] Pereira M, Matuszewska K, Jamieson C, Petrik J (2021) Characterizing endocrine status, tumor hypoxia and immunogenicity for therapy success in epithelial ovarian cancer. Front Endocrinol 12:772349–772349. 10.3389/fendo.2021.772349 10.3389/fendo.2021.772349 PMC8635771 34867818

[CR27] Pokhriyal R, Hariprasad R, Kumar L, Hariprasad G (2019) Chemotherapy resistance in advanced ovarian cancer patients. Biomark Cancer 11:1179299x19860815. 10.1177/1179299x19860815 31308780 10.1177/1179299X19860815 PMC6613062

[CR28] Romani A, Casciano F, Stevanin C, Maietti A, Tedeschi P, Secchiero P, Marchetti N, Voltan R (2021) Anticancer activity of aqueous extracts from *Asparagus officinalis* L. byproduct on breast cancer cells. Molecules 26(21):6369 34770777 10.3390/molecules26216369 PMC8588164

[CR29] Russell FM, Hardie DG (2020) AMP-activated protein kinase: do we need activators or inhibitors to treat or prevent cancer? Int J Mol Sci 22(1):186 33375416 10.3390/ijms22010186 PMC7795930

[CR30] Safinya CR, Chung PJ, Song C, Li Y, Ewert KK, Choi MC (2016) The effect of multivalent cations and Tau on paclitaxel-stabilized microtubule assembly, disassembly, and structure. Adv Colloid Interface Sci 232:9–16. 10.1016/j.cis.2015.11.002 26684364 10.1016/j.cis.2015.11.002 PMC4864139

[CR31] Shi X, Sun X (2017) Regulation of paclitaxel activity by microtubule-associated proteins in cancer chemotherapy. Cancer Chemother Pharmacol 80(5):909–917. 10.1007/s00280-017-3398-2 28741098 10.1007/s00280-017-3398-2

[CR32] Siegel RL, Miller KD, Fuchs HE, Jemal A (2022) Cancer statistics, 2022. CA Cancer J Clin 72(1):7–33. 10.3322/caac.21708 35020204 10.3322/caac.21708

[CR33] Srinivas US, Tan BWQ, Vellayappan BA, Jeyasekharan AD (2019) ROS and the DNA damage response in cancer. Redox Biol 25(101084):21 10.1016/j.redox.2018.101084 PMC6859528 30612957

[CR34] Subramaniam S, Selvaduray KR, Radhakrishnan AK (2019) Bioactive compounds: natural defense against cancer? Biomolecules 9(12):758 31766399 10.3390/biom9120758 PMC6995630

[CR35] Sugiyama A, Ohta T, Obata M, Takahashi K, Seino M, Nagase S (2020) xCT inhibitor sulfasalazine depletes paclitaxel-resistant tumor cells through ferroptosis in uterine serous carcinoma. Oncol Lett 20(3):2689–2700 32782585 10.3892/ol.2020.11813 PMC7400102

[CR36] Tan MM, Chen MH, Han F, Wang JW, Tu YX (2021) Role of bioactive constituents of *Panax notoginseng* in the modulation of tumorigenesis: a potential review for the treatment of cancer. Front Pharmacol 12:738914. 10.3389/fphar.2021.738914 34776959 10.3389/fphar.2021.738914 PMC8578715

[CR37] Tendulkar S, Dodamani S (2021) Chemoresistance in ovarian cancer: prospects for new drugs. Anticancer Agents Med Chem 21(6):668–678. 10.2174/1871520620666200908104835 32900355 10.2174/1871520620666200908104835

[CR38] Tropé C, Kaern J, Kristensen G, Rosenberg P, Sorbe B (1997) Paclitaxel in untreated FIGO stage III suboptimally resected ovarian cancer. Ann Oncol 8(8):803–806. 10.1023/a:1008230909599 9332691 10.1023/a:1008230909599

[CR39] Turrini E, Ferruzzi L, Fimognari C (2014) Natural compounds to overcome cancer chemoresistance: toxicological and clinical issues. Expert Opin Drug Metab Toxicol 10(12):1677–1690 25339439 10.1517/17425255.2014.972933

[CR40] Tymon-Rosario J, Adjei NN, Roque DM, Santin AD (2021) Microtubule-interfering drugs: current and future roles in epithelial ovarian cancer treatment. Cancers. 10.3390/cancers13246239 34944858 10.3390/cancers13246239 PMC8699494

[CR41] Vaidyanathan A, Sawers L, Gannon AL, Chakravarty P, Scott AL, Bray SE, Ferguson MJ, Smith G (2016) ABCB1 (MDR1) induction defines a common resistance mechanism in paclitaxel- and olaparib-resistant ovarian cancer cells. Br J Cancer 115(4):431–441 27415012 10.1038/bjc.2016.203 PMC4985349

[CR42] Wang H, Ng TB (2001) Isolation of a novel deoxyribonuclease with antifungal activity from *Asparagus officinalis* seeds. Biochem Biophys Res Commun 289(1):120–124 11708787 10.1006/bbrc.2001.5963

[CR43] Wang J, Liu Y, Zhao J, Zhang W, Pang X (2013) Saponins extracted from by-product of *Asparagus officinalis* L. suppress tumour cell migration and invasion through targeting Rho GTPase signalling pathway. J Sci Food Agric 93(6):1492–1498. 10.1002/jsfa.5922 23450726 10.1002/jsfa.5922

[CR44] Xiang J, Xiang Y, Lin S, Xin D, Liu X, Weng L, Chen T, Zhang M (2014) Anticancer effects of deproteinized asparagus polysaccharide on hepatocellular carcinoma in vitro and in vivo. Tumour Biol 35(4):3517–3524. 10.1007/s13277-013-1464-x 24310501 10.1007/s13277-013-1464-x

[CR45] Xie S, Ogden A, Aneja R, Zhou J (2016) Microtubule-binding proteins as promising biomarkers of paclitaxel sensitivity in cancer chemotherapy. Med Res Rev 36(2):300–312 26332739 10.1002/med.21378 PMC4778546

[CR46] Xu G, Kong W, Fang Z, Fan Y, Yin Y, Sullivan SA, Tran AQ, Clark LH, Sun W, Hao T, Zhao L, Zhou C, Bae-Jump VL (2021a) *Asparagus officinalis* exhibits anti-tumorigenic and anti-metastatic effects in ovarian cancer. Front Oncol 11:688461 34336674 10.3389/fonc.2021.688461 PMC8317209

[CR47] Xu G, Kong W, Fang Z, Fan Y, Yin Y, Sullivan SA, Tran AQ, Clark LH, Sun W, Hao T, Zhao L, Zhou C, Bae-Jump VL (2021b) *Asparagus officinalis* exhibits anti-tumorigenic and anti-metastatic effects in ovarian cancer. Front Oncol 11:688461. 10.3389/fonc.2021.688461 34336674 10.3389/fonc.2021.688461 PMC8317209

[CR48] Yan YB, Tian Q, Zhang JF, Xiang Y (2020) Antitumor effects and molecular mechanisms of action of natural products in ovarian cancer. Oncol Lett 20(5):141. 10.3892/ol.2020.12001 32934709 10.3892/ol.2020.12001 PMC7471673

[CR49] Yang Q, Huang J, Wu Q, Cai Y, Zhu L, Lu X, Chen S, Chen C, Wang Z (2014) Acquisition of epithelial-mesenchymal transition is associated with Skp2 expression in paclitaxel-resistant breast cancer cells. Br J Cancer 110(8):1958–1967 24642627 10.1038/bjc.2014.136 PMC3992499

[CR50] Zhang ZH, Fan ST, Huang DF, Yu Q, Liu XZ, Li C, Wang S, Xiong T, Nie SP, Xie MY (2018a) Effect of Lactobacillus plantarum NCU116 fermentation on *Asparagus officinalis* polysaccharide: characterization, antioxidative, and immunoregulatory activities. J Agric Food Chem 66(41):10703–10711 30251849 10.1021/acs.jafc.8b03220

[CR51] Zhang W, He W, Shi X, Li X, Wang Y, Hu M, Ma F, Tao N, Wang G, Qin Z (2018b) An Asparagus polysaccharide fraction inhibits MDSCs by inducing apoptosis through toll-like receptor 4. Phytother Res 32(7):1297–1303. 10.1002/ptr.6058 29532545 10.1002/ptr.6058

[CR52] Zhang F, Zhang YY, Sun YS, Ma RH, Thakur K, Zhang JG, Wei ZJ (2020) Asparanin A from *Asparagus officinalis* L. induces G0/G1 Cell cycle arrest and apoptosis in human endometrial carcinoma ishikawa cells via mitochondrial and PI3K/AKT signaling pathways. J Agric Food Chem 68(1):213–224. 10.1021/acs.jafc.9b07103 31861958 10.1021/acs.jafc.9b07103

[CR53] Zhang F, Ni ZJ, Ye L, Zhang YY, Thakur K, Cespedes-Acuña CL, Han J, Zhang JG, Wei ZJ (2021a) Asparanin A inhibits cell migration and invasion in human endometrial cancer via Ras/ERK/MAPK pathway. Food Chem Toxicol 150:112036. 10.1016/j.fct.2021.112036 33561516 10.1016/j.fct.2021.112036

[CR54] Zhang F, Ni ZJ, Ye L, Zhang YY, Thakur K, Cespedes-Acuña CL, Han J, Zhang JG, Wei ZJ (2021b) Asparanin A inhibits cell migration and invasion in human endometrial cancer via Ras/ERK/MAPK pathway. Food Chem Toxicol 150(112036):6 10.1016/j.fct.2021.112036 33561516

