# Supplementary material for: Asparagus officinalis combined with paclitaxel exhibited synergistic anti-tumor activity in paclitaxel-sensitive and -resistant ovarian cancer cells
Source: J Cancer Res Clin Oncol. 2022 Aug 25;149(7):3871–83. doi: 10.1007/s00432-022-04276-8 (PMC10314877; doi:10.1007/s00432-022-04276-8)
Supplement: Supplementary file 1 — Supplementary file1 (DOCX 37 KB) [file 432_2022_4276_MOESM1_ESM.docx]

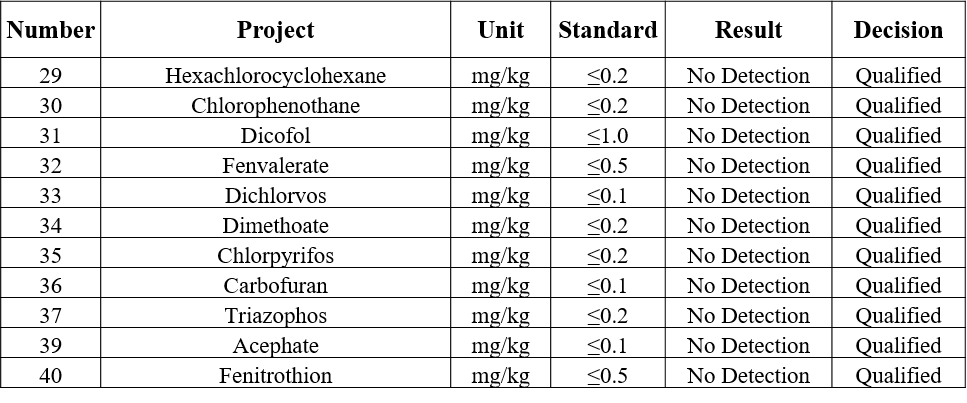


**Supplemental table 1. Pesticide test report***

* The original ASP test report was written in Chinese. This table is a partial translation of the ASP's original test report, showing only the results for the pesticides in the ASP. Authors will provide original test reports upon request by editors and reviewers. The pesticides ingredients were detected by chromatographic methods.
