# Supplementary material for: Asparagus officinalis combined with paclitaxel exhibited synergistic anti-tumor activity in paclitaxel-sensitive and -resistant ovarian cancer cells
Source: J Cancer Res Clin Oncol. 2022 Aug 25;149(7):3871–83. doi: 10.1007/s00432-022-04276-8 (PMC10314877; doi:10.1007/s00432-022-04276-8)
Supplement: Supplementary file 2 — Supplementary file2 (PDF 6993 KB) [file 432_2022_4276_MOESM2_ESM.pdf]

出厂检验报告

企业名称:菏泽巨鑫源食品有限公司芦笋生物科技分公司　　报告编号:JXY-YP-2-180501

样品名称	芦笋浓缩汁	数　量	200Kg/桶
生产日期	2018.4.16	检验日期	2018.4.25
班次	白班	检验依据	GB/T 31121

检 验 结 果 表

序号	检验项目	单位	技术要求	检验结果	单项判定
1	感观	/	色泽：色泽均匀呈乳状<br>气味：具有芦笋特有的气味，无异味。<br>杂质：无肉眼可见外来杂质。	正常	合格
2	净含量	Kg	约 200	200	符合
3	可溶性固形物	%	60-68	62	合格
4	PH 值	/	3.6-5.6	4.48	合格
5	菌落总数	cfu/ ml	≤100	11	符合
6	大肠菌群	MPN/100 ml	≤3	0	符合
7	霉菌	cfu/ ml	≤10	2	符合
8	酵母菌	cfu/ m	≤10	3	符合
9	沙门氏菌	/25g	n=5，c=0,m=0	未检出	合格
10	金黄色葡萄球菌	Cfu/ml	n=5，c=1,m=100，M=1000	<10/<10/<10/<10/<10	合格
11	苯甲酸及其钠盐（以苯甲酸计）	g/kg	≤1.0	未检出	合格
12	二氧化硫残留量	g/kg	≤0.05	0.02	合格
13	山梨酸及其钾盐（以山梨酸计）	g/kg	≤2.0	未检出	合格
14	糖精钠（以糖精计）	g/kg	不得检出	未检出	合格
15	安赛蜜	g/kg	≤0.3	未检出	合格
16	甜蜜素（以环己基氨基磺酸计）	g/kg	≤0.65	未检出	合格
17	柠檬黄及其铝色淀（以柠檬黄计）	g/kg	≤0.1	未检出	合格
18	日落黄及其铝色淀（以日落黄计）	g/kg	≤0.1	未检出	合格
19	亮蓝及其铝色淀（以亮蓝计）	g/kg	≤0.025	未检出	合格

20	胭脂红及其铝色淀（以胭脂红计）	g/kg	≤0.05	未检出	合格
21	苋菜红及其铝色淀（以苋菜红计）	g/kg	≤0.05	未检出	合格
22	总砷（以 As 计）	mg/kg	不得检出	未检出	合格
23	铅（以 Pb 计）	mg/L	≤0.05	未检出	合格
24	镉（以 Cd 计）	mg/kg	≤0.5	未检出	合格
25	铁	mg/L	≤15	5.0	合格
26	铜	mg/L	≤5	3.9	合格
27	锡	mg/L	200	<20.0	合格
28	锌	mg/L	≤5	1.5	合格
29	六六六	mg/kg	≤0.2	未检出	合格
30	滴滴涕	mg/kg	≤0.2	未检出	合格
31	三氯杀螨醇	mg/kg	≤1.0	未检出	合格
32	氰戊菊酯	mg/kg	≤0.5	未检出	合格
33	敌敌畏	mg/kg	≤0.1	未检出	合格
34	乐果	mg/kg	≤0.2	未检出	合格
35	毒死蜱	mg/kg	≤0.2	未检出	合格
36	克百威	mg/kg	≤0.1	未检出	合格
37	三唑磷	mg/kg	≤0.2	未检出	合格
39	乙酰甲胺磷	mg/kg	≤0.1	未检出	合格
40	杀螟硫磷	mg/kg	≤0.5	未检出	合格
41	标签	/	符合 GB7718-2011 的要求	符合要求	合格
检验结论	该产品所检项目 符合 GB/T 31121 标准要求。<br>检验专章<br>报告日期： 2018 年 5 月 5 日				

审核人： 李子恩

主检人：马洋
